# Sperm IZUMO1 Is Required for Binding Preceding Fusion With Oolemma in Mice and Rats

**DOI:** 10.3389/fcell.2021.810118

**Published:** 2022-01-12

**Authors:** Takafumi Matsumura, Taichi Noda, Yuhkoh Satouh, Akane Morohoshi, Shunsuke Yuri, Masaki Ogawa, Yonggang Lu, Ayako Isotani, Masahito Ikawa

**Affiliations:** ^1^ Department of Experimental Genome Research, Research Institute for Microbial Diseases, Osaka University, Suita, Japan; ^2^ Graduate School of Pharmaceutical Sciences, Osaka University, Suita, Japan; ^3^ Division of Reproductive Biology, Institute of Resource Development and Analysis, Kumamoto University, Kumamoto, Japan; ^4^ Priority Organization for Innovation and Excellence, Kumamoto University, Kumamoto, Japan; ^5^ Graduate School of Medicine, Osaka University, Suita, Japan; ^6^ Graduate School of Science and Technology, Nara Institute of Science and Technology, Ikoma, Japan; ^7^ Immunology Frontier Research Center, Osaka University, Suita, Japan; ^8^ Laboratory of Reproductive Systems Biology, Institute of Medical Science, The University of Tokyo, Tokyo, Japan

**Keywords:** fertilization, sperm-oolemma adhesion, gamete fusion, FOLR4, knockout, Juno

## Abstract

Fertilization occurs as the culmination of multi-step complex processes. First, mammalian spermatozoa undergo the acrosome reaction to become fusion-competent. Then, the acrosome-reacted spermatozoa penetrate the zona pellucida and adhere to and finally fuse with the egg plasma membrane. IZUMO1 is the first sperm protein proven to be essential for sperm-egg fusion in mammals, as *Izumo1* knockout mouse spermatozoa adhere to but fail to fuse with the oolemma. However, the IZUMO1 function in other species remains largely unknown. Here, we generated *Izumo1* knockout rats by CRISPR/Cas9 and found the male rats were infertile. Unlike in mice, *Izumo1* knockout rat spermatozoa failed to bind to the oolemma. Further investigation revealed that the acrosome-intact sperm binding conceals a decreased number of the acrosome-reacted sperm bound to the oolemma in *Izumo1* knockout mice. Of note, we could not see any apparent defects in the binding of the acrosome-reacted sperm to the oolemma in the mice lacking recently found fusion-indispensable genes, *Fimp*, *Sof1*, *Spaca6*, or *Tmem95*. Collectively, our data suggest that IZUMO1 is required for the sperm-oolemma binding prior to fusion at least in rat.

## Introduction

In sexual reproduction, the binding and fusion between the plasma membranes of spermatozoa and eggs are fundamental prerequisites for successful fertilization. In mammals, the spermatozoa ejaculated into the female reproductive tract undergo physiological, biochemical, and morphological changes (i.e., capacitation and acrosome reaction) to gain fertilization competency. The capacitated and acrosome-reacted sperm pass through the oocyte cumulus cell layer and the zona pellucida (ZP) and eventually bind to and fuse with the egg plasma membrane (Okabe, 2013). In the early days, several candidate sperm proteins involved in sperm-egg fusion have been identified using enzyme inhibitors and antibodies that block the *in vitro* fertilization (IVF) (Okabe, 2018). However, when their functions were challenged using the gene knockout (KO) mice, only IZUMO1 was found indispensable for the sperm-egg fusion (Okabe, 2018; Fujihara et al., 2020; Lamas-Toranzo et al., 2020; Noda et al., 2020).

IZUMO1 is an immunoglobulin (Ig)-like type I membrane protein that localizes to the acrosomal membrane and translocates to the sperm plasma membrane during the acrosome reaction (Satouh et al., 2012; Isotani et al., 2017). *Izumo1* KO spermatozoa can penetrate the ZP but cannot fuse with the egg plasma membrane, resulting in male sterility (Inoue et al., 2005). Through an Avidity-based Extracellular Interaction Screen (AVEXIS) assay, IZUMO1 was found to interact with a glycosylphosphatidylinositol (GPI)-anchored protein, JUNO (also called FOLR4 or IZUMO1R), expressed on the oolemma (Bushell et al., 2008; Bianchi et al., 2014). The tertiary structures of IZUMO1, JUNO, and their complex have been intensively studied by X-ray crystallography (Aydin et al., 2016; Han et al., 2016; Kato et al., 2016; Nishimura et al., 2016; Ohto et al., 2016). The IZUMO1-JUNO, ligand-receptor interaction exists in mice and humans in a species-specific manner, implying that the two molecules might govern the gamete incompatibility among species (Bianchi and Wright, 2015).

Nevertheless, unlike other eukaryotic fusion proteins (e.g., HAP2/GCS1), neither of the two molecules possesses a fusogenic peptide or enables the cell-cell fusion *in vitro* by transient expression (Bianchi et al., 2014; Aydin et al., 2016). In addition, the cultured cells, such as HEK293T and COS-7 cells, transiently expressing mouse IZUMO1 can tightly bind to but not fuse with the ZP-free mouse eggs, suggesting the IZUMO1-JUNO interaction alone is insufficient for membrane fusion (Inoue et al., 2013; Ohto et al., 2016). Given these findings, researchers speculate that IZUMO1 and JUNO are implicated in sperm-egg binding rather than fusion. However, the number of *Izumo1* KO mouse spermatozoa bound to the oolemma of the ZP-free eggs is comparable to the wild-type group (Inoue et al., 2005), rendering the detailed role of IZUMO1 during the sperm-egg fusion unclear.

In this study, we deleted *Izumo1* in rats using the CRISPR/Cas9 system and analyzed the IZUMO1 function during rat fertilization. Combined with a reanalysis of the *Izumo1* KO mice, our data indicate that IZUMO1 is required for binding the acrosome-reacted spermatozoa to oolemma prior to fusion.

## Materials and Methods

### Animals

All mice and rats were maintained under specific-pathogen-free conditions with *ad libitum* feeding. B6D2F1 mice were purchased from Japan SLC, Inc. B6D2-Tg(CAG/Su9-DsRed2,Acr3-EGFP)RBGS002Osb mice and STOCK Izumo1<tm1Osb> (*Izumo1* KO or *Izumo* ^*−/−*^) mice that are generated and maintained in our laboratory are used for generating *Izumo1* ^*−/−*^; RBGS mice. B6D2-4930451I11Rik<em1Osb> (*Fimp* KO) mice, STOCK Llcfc1<em2Osb> (*Sof1* KO) mice, B6D2-Spaca6<em2Osb> (*Spaca6* KO) mice, and B6D2-Tmem95<em1Osb> (*Tmem95* KO) mice are generated and maintained in our laboratory. WIAR rats were purchased from Institute for Animal Reproduction. Wistar rats were purchased from Charles River Laboratories Japan, Inc**.**

### Protein Sequence Alignment and Prediction of Domain Structure

IZUMO1 sequences used for alignment are retrieved from the National Center for Biotechnology Information (NCBI) database: *Homo sapiens* (Human), NP_872381.2; *Mus musculus* (Mouse), NP_001018013.1; *Rattus norvegicus* (Rat), NP_001017514.1; *Bos taurus* (Cattle), XP_024835010.1; *Strigops habroptila* (Kakapo), XP_030366609.1; *Terrapene carolina triunguis* (Turtle), XP_026513927.1; and *Danio rerio* (Zebrafish), NP_001314727.1. The domain structure of IZUMO1 was derived from the NCBI database or was predicted by the Simple Modular Architecture Research Tool (SMART). Sequence alignment of IZUMO1 homologs was conducted and visualized using the Clustal Omega program (https://www.ebi.ac.uk/Tools/msa/clustalo/).

### Generation of *Izumo1*-Deficient Rat

We generated a pX330 (#42230, Addgene, Cambridge, MA, USA) plasmid encoding a single-guide RNA (sgRNA) and the Cas9 protein to target the second exon of *Izumo1* (target sequence: 5′-GGT​GGC​TGC​AAT​AAA​GAC​TT-3′). The DNA cleavage activity of this plasmid was validated as described previously (Mashiko et al., 2013). Superovulated WIAR (3–4 weeks old) females were mated with the mature males, and the two-pronuclear zygotes were collected from the oviduct. The circular pX330 plasmid was injected into one pronucleus of each zygote at concentrations of 5 ng/μl, 10 ng/μl, or 20 ng/μl (Mashiko et al., 2013). The following primer set was used for genotyping PCR of rat *Izumo1* (Forward: 5′-TGT​TTA​GCA​CCC​TCC​TCC​CTG​C-3′, Reverse: 5′-AGA​GAG​TAT​GTA​TCT​CTG​CCT​GTC​CTG​G-3′).

### RT-PCR

The total RNA was collected from the adult rat testes, purified using NucleoSpin RNA Plus (Takara, Kyoto, Japan), and reverse-transcribed into cDNA using SuperScript IV VILO Master Mix (Thermo Fisher Scientific, Waltham, MA, USA). PCR was performed using KOD DNA polymerase (Toyobo, Osaka, Japan); the PCR conditions for detecting rat *Izumo1* were as follows: initial denaturing at 98°C for 30 s, followed by denaturing at 98°C for 15 s, annealing at 55°C for 5 s, and elongation 68°C for 5 s for 33 cycles. The PCR conditions for detecting rat *Actb* were as follows: initial denaturing at 98°C for 30 s, followed by denaturing at 98°C for 15 s, annealing at 58°C for 5 s, and elongation at 68°C for 5 s for 30 cycles. The PCR primers for rat *Izumo1* were: Forward; 5′-ATG​GGG​CTA​CAT​TTT​ACA​CTC-3′ and Reverse; 5′-TTA​GTT​ATC​TGC​TTC​CTC​AAC​C-3’. The PCR primers for rat *Actb* were: Forward; 5′-GTC​CAC​CCG​CGA​GTA​CAA​C-3′ and Reverse; 5′-GGA​TGC​CTC​TCT​TGC​TCT​GG-3′.

### Antibodies

The mouse monoclonal antibody against rat CD46 [MM1] (ab180652) was purchased from Abcam (Cambridge, UK). The rat monoclonal antibody against IZUMO1 (KS-64 125) (Ikawa et al., 2011) and rabbit polyclonal antibody against human IZUMO1 (Inoue et al., 2005) are generated in our laboratory as previously described. The goat anti-rat IgG Alexa Fluor 488-conjugated antibody (A11006), goat anti-mouse IgG antibody labeled with Alexa Fluor 568 (A11004), and goat anti-rabbit IgG Alexa Fluor Plus 488-conjugated antibody (A11034) were purchased from Thermo Fisher Scientific.

### Testis Histological Analysis

Testes were fixed in 4% paraformaldehyde in PBS and were processed for plastic embedding using Technovit^®^ 8100 (Mitsui Chemicals, Tokyo, Japan) as described previously (Matsumura et al., 2019). Sections were made at a thickness of 5 µm and treated with 1% periodic acid (Wako, Osaka, Japan) for 10 min, followed by treatment with the Schiff’s reagent (Muto Pure Chemicals Co., Ltd., Tokyo, Japan) for 15 min. The sections were further stained with Mayer’s hematoxylin solution (Wako) and observed under a light microscope.

### Sperm Analysis

For detecting IZUMO1 in sperm cells, the frozen rat testes were disaggregated in PBS using BioMasher II (Nippi, Tokyo, Japan), and the germ cells were smeared on glass slides. The cells were air-dried on microscope slides, washed with PBS, and permeabilized with methanol. After treatment with image-iT FX Signal enhancer (Thermo Fisher Scientific) for 30 min at room temperature, the cells were incubated with the rabbit anti-human IZUMO1 polyclonal antibody (1:100) diluted by Can Get Signal A (Toyobo) at 4°C for overnight. After incubation with goat anti-rabbit IgG Alexa Fluor Plus 488 (1:200) and Lectin PNA Alexa Fluor 568 conjugate (Thermo Fisher Scientific) (1:200) for 1 h at room temperature, the spermatozoa were stained with Hoechst 33342 to visualize the nuclei (Dojindo, Kumamoto, Japan) and were mounted in Prolong Diamond (Thermo Fisher Scientific). For sperm morphological analysis, the cauda epididymal spermatozoa of male rats were incubated in the Human Tubal Fluid (HTF) medium (Aoto et al., 2011) for 10 min and observed under a BX50F phase-contrast microscope (Olympus, Tokyo, Japan).

### Mating Test

Each male rat was caged with two Wistar females for 2 months. The number of delivered pups was counted. Copulation was confirmed by the formation of vaginal plugs or the presence of spermatozoa in the vaginal smears. Alternatively, superovulated Wistar females (3–4 weeks old) were mated with the male rats (3–6 months old). The eggs were collected from the oviducts of the superovulated females about 22 h after the hCG injection and mating. The eggs were observed under an inverted microscope (Olympus IX71), and the fertilization rate was recorded.

### Rat Sperm-Oolemma Fusion Assay

The HTF medium was prepared as described previously (Aoto et al., 2011). The cauda epididymal spermatozoa from male rats (3–6 months old) were preincubated for an hour in the HTF medium. The final sperm concentration was adjusted to 1 × 10^5^ spermatozoa/mL in 100 µl HTF medium drops for insemination. The cumulus-oocyte complexes were collected from the oviductal ampulla of the superovulated females (3–4 weeks old), and the cumulus cells were removed by 0.03% hyaluronidase. The cumulus-free eggs were subsequently treated with 1 mg/ml collagenase to remove the ZP. To assess the sperm-egg fusion, the ZP-free eggs were fixed with 2% paraformaldehyde and stained by Hoechst 33342 after insemination for 5 h. Swollen sperm nuclei could be observed in the ooplasm when fusion occurred. The sperm-oolemma adhesion was assessed under a microscope at 0, 0.5, 2, and 5 h after insemination. To examine the acrosome status, spermatozoa were probed with anti-CD46 antibody (MM1) (1:200) and goat anti-mouse IgG Alexa Fluor 568-conjugated secondary antibody (1:200) in the HTF medium. An hour after insemination, the eggs were stained by Hoechst 33342, and the Z-stack images were captured using a BZ-X710 microscope (Keyence, Osaka, Japan). The total number of bound spermatozoa was counted. The spermatozoa in the HTF medium were smeared on microscope slides and observed under a fluorescence microscope.

### Mouse Sperm-Oolemma Fusion Assay

The Toyoda-Yokoyama-Hosi (TYH) medium was prepared as described previously (Toyoda et al., 1971) and used for sperm preincubation, egg preparation, and insemination. The cauda epididymal spermatozoa from adult male mice (3–6 months) were preincubated for 2 h. The final concentration of spermatozoa was adjusted to 1 × 10^5^ spermatozoa/mL in 100 µl TYH medium drops for insemination. Twenty minutes after insemination, the ZP-free eggs were moved to fresh medium drops, fixed with 0.8% paraformaldehyde, and stained by Hoechst 33342. The eggs were transferred to glass-bottomed chambers to observe *Acr-*EGFP fluorescence using a spinning-disk confocal microscope (Olympus). The cauda epididymal spermatozoa obtained from *Fimp*, *Sof1*, *Tmem95*, and *Spaca6* KO male mice were preincubated at a concentration of 1–2 × 10^5^ spermatozoa/mL for 2.5 h followed by probing with the monoclonal antibody against IZUMO1 (KS-64 125) (1:100) and goat anti-rat IgG Alexa Fluor 488-conjugated antibody (1:200) for another 30 min. The sperm concentration was adjusted to 1 × 10^5^ spermatozoa/mL in 100 µl fresh medium drops and incubated with the ZP-free eggs for 30 min. The eggs were gently washed in fresh medium drops three times and fixed with 0.2% paraformaldehyde. The spermatozoa from B6D2F1 or *Sof1* ^*+/−*^(*Sof1* Het) male mice were used as control. The eggs were observed using a BZ-X710 microscope (Keyence). For calculating the total number of spermatozoa bound to the oolemma, the sperm tails were counted using a bright field microscope. The spermatozoa with and without green fluorescence in the acrosome were recognized as acrosome-intact and acrosome-reacted, respectively.

### Statistical Analysis

Statistical analyses were performed using a two-tailed student’s t-test (n ≧ 3). Differences were considered significant at *p* < 0.05. Data represent the means ± standard deviation (SD), and error bars indicate SD.

### Biological Resources Availability Statement

The *Izumo1* KO rat strain was deposited under the name W; WIAR-*Izumo1*<em1Osb>, which will be available through the National BioResource Project for the Rat in Japan (NBRP; Kyoto, Japan). The B6D2-Tg(CAG/Su9-DsRed2, Acr3-EGFP)RBGS002Osb mice, STOCK Izumo1<tm1Osb> (*Izumo1* KO or *Izumo1* ^*−/−*^) mice, B6D2-4930451I11Rik<em1Osb> (*Fimp* KO), STOCK Llcfc1<em2Osb> (*Sof1* KO), B6D2-Spaca6<em2Osb> (*Spaca6* KO), and B6D2-Tmem95<em1Osb> (*Tmem95* KO) mouse lines used in this study are available through either the Riken BioResource Center (Riken BRC; Tsukuba, Japan) or the Center for Animal Resources and Development, Kumamoto University (CARD; Kumamoto, Japan).

## Results

### *Izumo1* KO Male Rats Are Infertile due to Fertilization Defects

**FIGURE 1:**
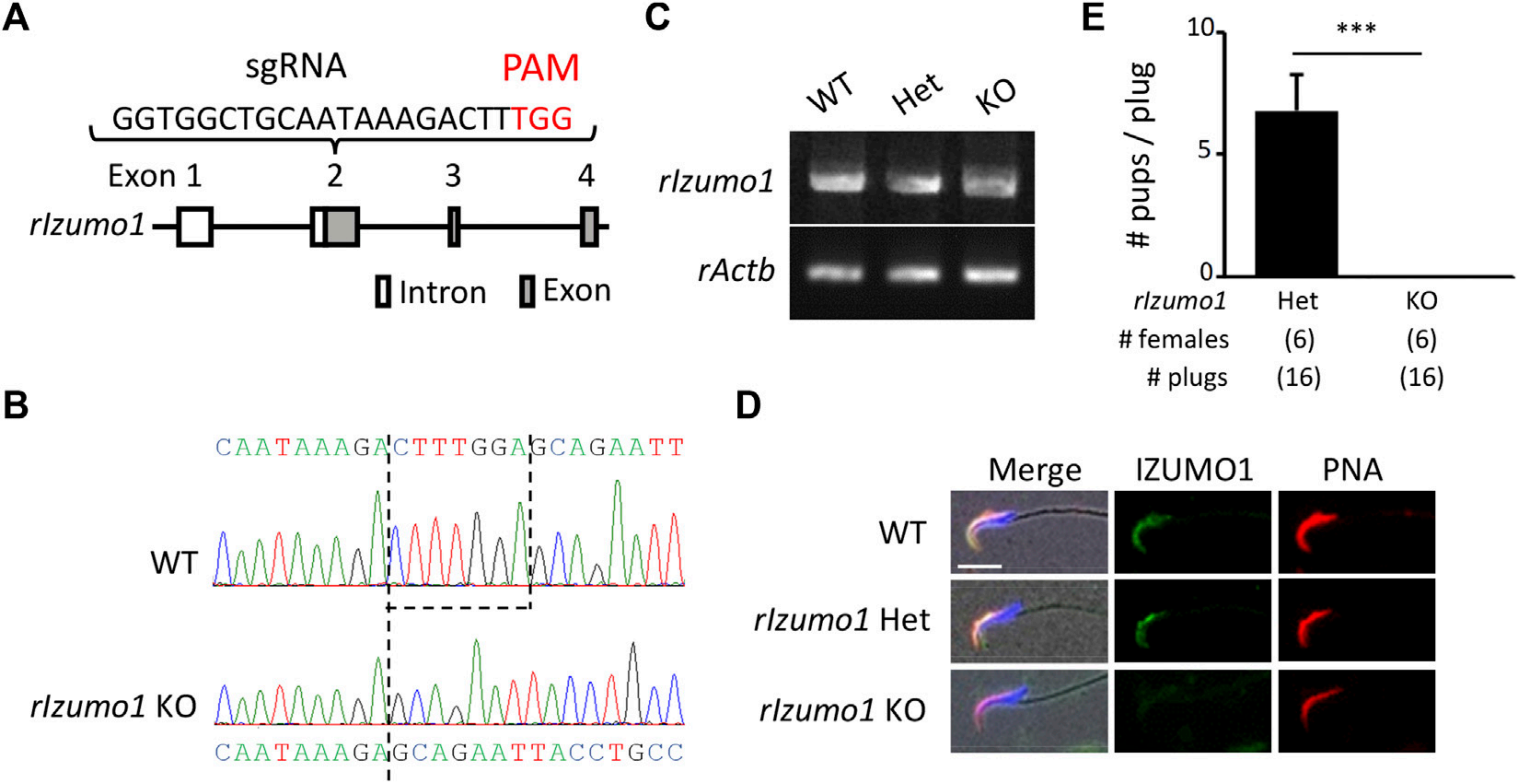
Generation of *Izumo1*-deficient rats using CRISPR/Cas9. **(A)** Design of sgRNA for disrupting *Izumo1* in rats. White and gray boxes represent the untranslated and coding regions, respectively. **(B)** Genomic *Izumo1* sequences of WT and mutant (*Izumo1* ^*−7*/*−7*^) alleles. **(C)** Detection of rat *Izumo1* mRNA in testis by RT-PCR. **(D)** Immunostaining of IZUMO1 in the testicular sperm from WT or mutant rats. The acrosomal caps and the nuclei were stained by the fluorophore-conjugated Lectin PNA and Hoechst 33342, respectively. Scale bar = 10 µm. **(E)** The average number of pups per copulatory plug. Three males each were tested for the *Izumo1* Het and *Izumo1* KO groups. ***p < 0.001.

The amino acid sequences of IZUMO1 are conserved in mammals, birds, reptiles, and fish (Supplementary Figure S1). To knockout rat *Izumo1* with the CRISPR/Cas9-mediated gene disruption, we designed a single-guide RNA (sgRNA) targeting the second exon of *Izumo1* (Figure 1A). By injecting the sgRNA/Cas9 expressing plasmid (pX330) into the pronuclei of 215 zygotes, we obtained a male and a female mutant rat (2/13 pups, 15.3%) (Supplementary Figures S2A,B). As the mutant male rat (*Izumo1* ^*−8*/*−29*^) showed sterility (Supplementary Figure 2C), we established a mutant line harboring 7-bp deletion by pairing the mutant female with a wild-type (WT) male (Figure 1B). We confirmed that *Izumo1* ^*−7/−7*^ (KO) rats that *Izumo1* mRNA derived from the mutant allele were transcribed in the testis (Figure 1C). But the translation of IZUMO1 is expected to be interrupted by a premature termination codon introduced by the frameshift (Supplementary Figure S2D). Indeed, the loss of IZUMO1 protein in the sperm acrosome was confirmed by immunostaining (Figure 1D). When *Izumo1* ^*wt*/−*7*^ (Het) or *Izumo1* KO male rats were individually caged with WT females for 2 months, all *Izumo1* Het males sired pups (pups/plug: 6.79 ± 1.45), whereas *Izumo1* KO males did not produce any pups despite the normal mating behavior indicated by formation of the copulation plugs (Figure 1E).

### *Izumo1* KO Rat Spermatozoa Show Impaired Adhesion to the Oolemma

**FIGURE 2:**
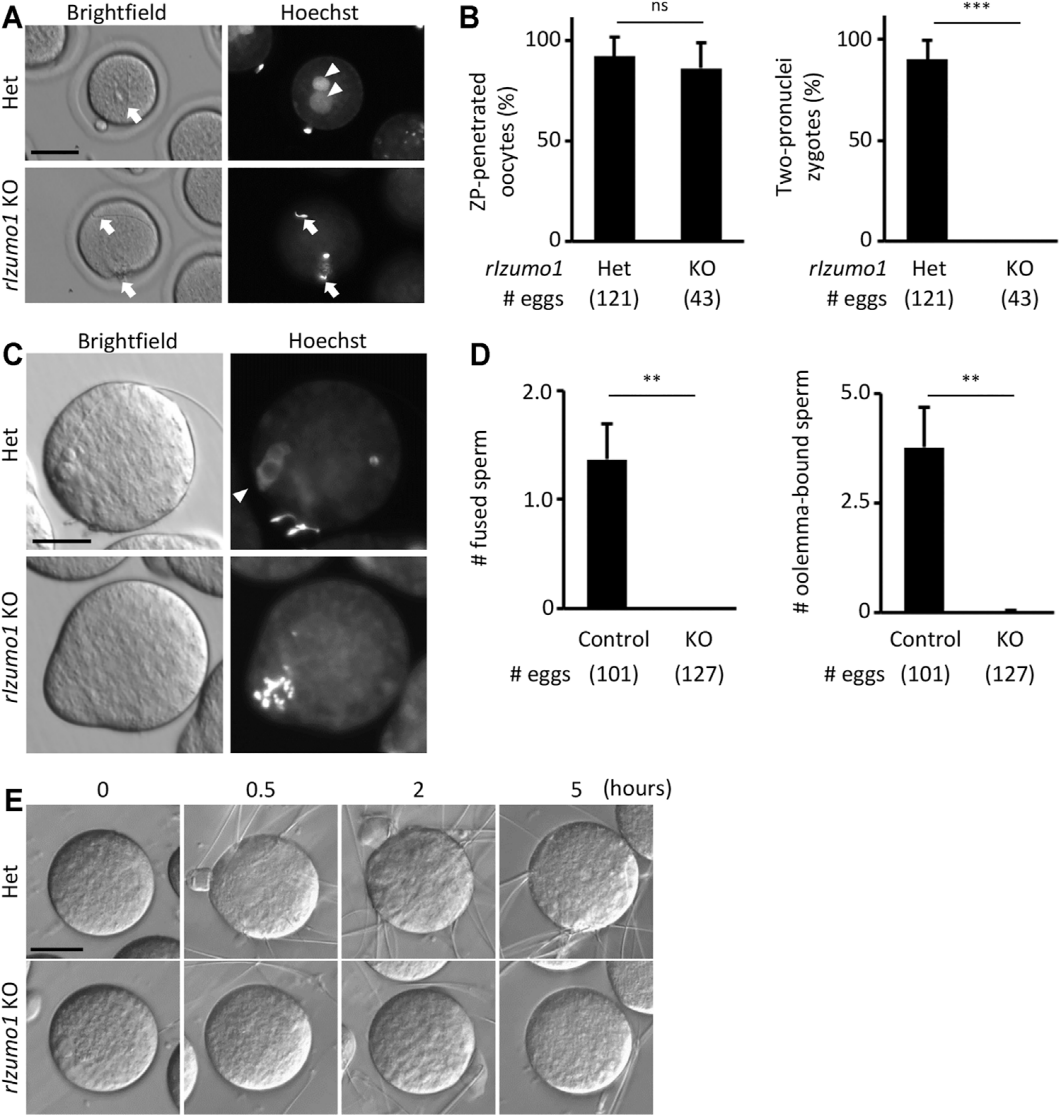
Impaired ability to bind to the oolemma in *Izumo1* KO rat sperm. **(A)** The eggs recovered from the ampulla of the superovulated females mated with *Izumo1* Het or *Izumo1* KO male. The nuclei were stained by Hoechst 33342. Scale bar = 100 µm. **(B)** The average ratio of the ZP-penetrated eggs (left) or two-pronuclear zygotes (right) to the total recovered eggs. ns represents no statistical difference. ***p < 0.001. **(C)** The sperm-egg fusion was assessed by staining the nuclei with Hoechst 33342. Fused sperm are indicated by the fluorescence signals of the swollen sperm nuclei. Scale bar = 50 µm. **(D)** The average numbers of the fused spermatozoa (left) and oolemma-bound spermatozoa (right). *Izumo1* WT and Het rat sperm were used as control. **p < 0.01 **(E)** The sperm-egg adhesion was captured at 0, 0.5, 2, and 5 h after insemination. Scale bar = 50 µm.

Periodic acid-Schiff (PAS) staining of testis sections showed no sign of defects in the spermatogenesis of *Izumo1* KO rats (Supplementary Figure S3A). In addition, there were no apparent abnormalities in the morphology of *Izumo1* KO spermatozoa collected from the cauda epididymis (Supplementary Figure S3B). To determine the fertilizing ability of *Izumo1* KO spermatozoa *in vivo*, we collected eggs from the oviduct of wild-type females 22 h after pairing with the KO males. Though *Izumo1* KO spermatozoa penetrated the ZP at a comparable rate as the Het spermatozoa, no fertilized eggs were observed (Figures 2A,B).

We next performed *in vitro* fertilization using the enzymatically ZP-removed eggs. While the WT and Het spermatozoa successfully adhered to and fused with the eggs after an hour of insemination (oolemma-bound and fused spermatozoa: 3.77 ± 0.94 and 1.36 ± 0.33 spermatozoa/egg, respectively), none of the *Izumo1* KO spermatozoa adhered to or fused with the eggs (oolemma-bound and fused spermatozoa: 0.02 ± 0.03 and 0 ± 0 spermatozoa/egg, respectively) (Figures 2C,D). Even when we extended the insemination period up to 5 h, adhesion of the *Izumo1* KO spermatozoa to the oolemma was not observed (Figure 2E, Supplementary Movies S1, S2). These findings suggest the sterility of *Izumo1* KO rats is attributed to the inability of KO spermatozoa to bind to the oolemma.

### Rat Spermatozoa Acquire Egg Binding Ability After Acrosome Reaction

In mice and hamsters, it is known that both the acrosome-intact and reacted spermatozoa can stick to the surface of the ZP-free eggs (Phillips and Yanagimachi, 1982; Calarco, 1991; Yanagimachi, 1994; Satouh et al., 2012). Since IZUMO1 is exposed to the sperm surface during the acrosome reaction (Satouh et al., 2012; Isotani et al., 2017), the attachment of the acrosome-intact spermatozoa to the oolemma does not involve the IZUMO1-JUNO interaction and does not lead to subsequent fusion between the two plasma membranes. Thus, in the following text, the acrosome-intact or reacted spermatozoa attachment to the oolemma is referred to as the fusion-incompetent or fusion-competent sperm adhesion, respectively. To rule out the fusion-incompetent sperm adhesion, we assessed the acrosome status of the live spermatozoa by immunostaining of CD46 (Supplementary Figure S4A). Like IZUMO1, CD46 is localized to the inner acrosomal membrane and is translocated to the sperm plasma membrane during the acrosome reaction (Mizuno et al., 2004). Since CD46 is inaccessible to the antibody before being exposed to the sperm surface, no fluorescence signals should be seen in the live acrosome-intact spermatozoa. As a result, 82.6 ± 12.4% of the spermatozoa adhered to the oolemma were acrosome-reacted, whereas 24.5 ± 11.2% of the spermatozoa in the medium were acrosome-reacted (Supplementary Figures S4B,C), indicating that the acrosome-intact spermatozoa are less prone to adhering to the oolemma compared with the acrosome-reacted spermatozoa in rats.

### IZUMO1 Plays a Critical Role in Acrosome Reacted Sperm Binding to Oolemma in Mice

**FIGURE 3:**
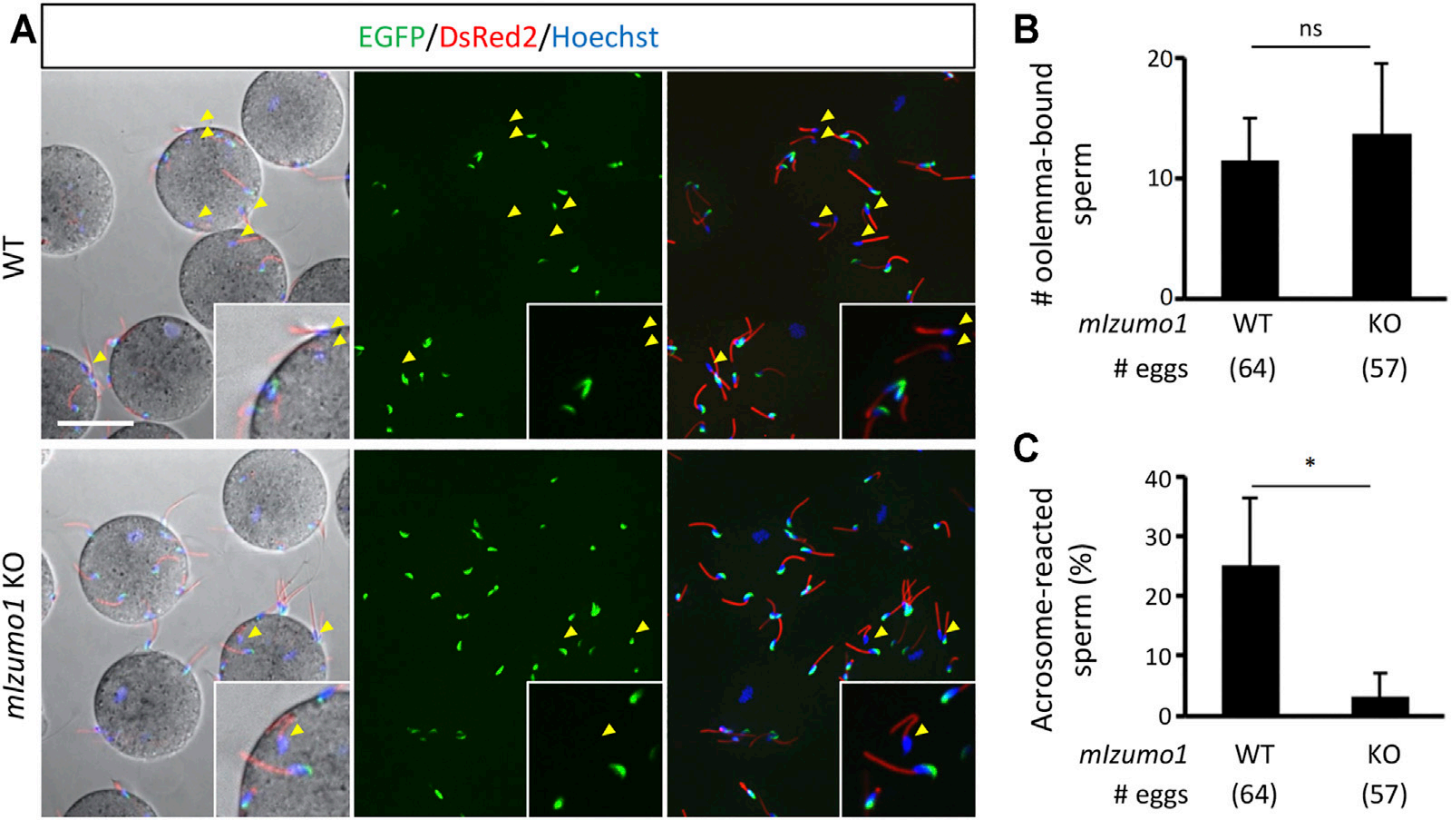
Reduced binding of the acrosome-reacted *Izumo1* KO mouse sperm to the oolemma. **(A)** A representative image of mouse sperm adhered to egg membrane. Spermatozoa with the EGFP signals are acrosome-intact, whereas the ones without EGFP are acrosome-reacted (yellow arrowhead). DsRed2 is expressed in the sperm midpiece. The sperm nuclei were stained by Hoechst 33342. Scale bar = 50 µm **(B)** The total numbers of oolemma-bound spermatozoa. ns represents no statistical difference. **(C)** The ratios of the acrosome-reacted sperm to the total number of oolemma-bound sperm. *p < 0.05.

The lack of adhesion ability of the *Izumo1* KO rat spermatozoa to the oolemma is distinct from our previous study, in which we could not see apparent defects in the adhesion of *Izumo1* KO mouse spermatozoa to the oolemma (Inoue et al., 2005). Of note, it was reported that the acrosome-reacted spermatozoa hardly bind to the *JUNO* KO eggs in mice (Bianchi et al., 2014). Thus, we speculated that an excessive number of the acrosome-intact spermatozoa adhered to the oolemma might conceal a lack of fusion-competent adhesion by the acrosome-reacted spermatozoa. To address this concern, we generated *Izumo1* KO mice carrying the RBGS (Red Body Green Spermatozoa) transgene that encodes EGFP in the acrosome and DsRed2 in the mitochondria to re-evaluate the sperm acrosome status during fertilization. The mouse spermatozoa were preincubated for 2 h and incubated with the ZP-free eggs for 20 min in the TYH medium. We did not see any differences in the average rates of the acrosome reaction in WT and *Izumo1* KO spermatozoa (WT: 20.8 ± 15.8%; KO: 21.7 ± 11.0%). While the total numbers of the spermatozoa bound to the oolemma were comparable between the WT and KO groups (Figures 3A,B), the percentage of the oolemma-bound acrosome-reacted spermatozoa was drastically decreased in the *Izumo1* KO mice (WT: 25.3 ± 11.3%; KO: 3.28 ± 3.78%, Figure 3C).

### Mouse Spermatozoa Lacking Other Fusion-Related Genes Are Not Defective in Binding to the Oolemma

**FIGURE 4:**
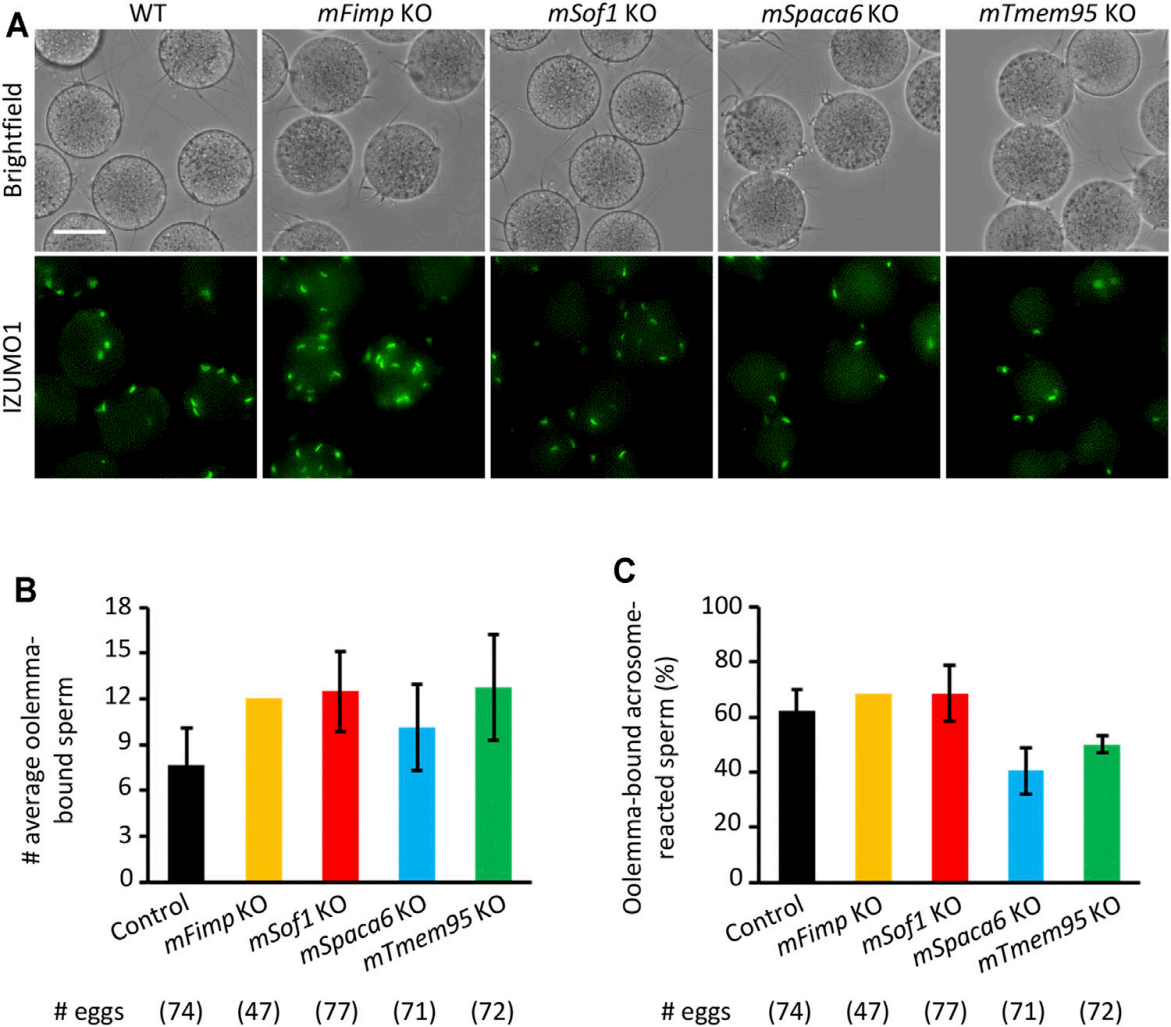
Mutant sperm lacking the novel fusion-related genes bind to the oolemma after the acrosome reaction. **(A**) Observation of the binding between WT eggs and WT or *Fimp*, *Sof1*, *Tmem95*, and *Spaca6* KO spermatozoa. The acrosome-reacted sperm on the oolemma are indicated by the fluorescence signals of IZUMO1. Scale bar = 50 µm. **(B)** The numbers of total oolemma-bound spermatozoa. WT and *Sof1* Het mouse sperm were used as control. **(C)** The ratio of the acrosome-reacted sperm to the total oolemma-bound sperm. WT and *Sof1* Het mouse sperm were used as control.

Recent studies uncovered novel sperm proteins necessary for sperm-egg fusion in addition to IZUMO1 (Lorenzetti et al., 2014; Fujihara et al., 2020; Lamas-Toranzo et al., 2020; Noda et al., 2020; Inoue et al., 2021; Noda et al., 2021), yet whether these molecules are involved in the gamete adhesion or fusion remains unclear. Hence, we performed the sperm-egg fusion assays using the *Fimp*, *Sof1*, *Spaca6*, and *Tmem95* mutant mouse spermatozoa with the sperm acrosome status being unraveled using an anti-IZUMO1 antibody. As a result, we could not see apparent differences in both the total numbers of bound spermatozoa and the ratios of the acrosome-reacted spermatozoa to the total bound spermatozoa between the WT and KO groups (Figures 4A–C).

## Discussion

The cell-cell membrane adhesion and fusion processes are considered independent biological events, each of which requires its suites of molecules. For instance, the gamete adhesion is potentiated by FUS1, whereas HAP2 regulates the gamete fusion in *Chlamydomonas reinhardtii* (Misamore et al., 2003; Liu et al., 2008; Aguilar et al., 2013; Hernandez and Podbilewicz, 2017). In mammal syngamy, studies using the cultured cell lines strongly suggest that IZUMO1 and JUNO are responsible for the sperm-egg adhesion instead of fusion per se – the HEK293T, COS-7, and K562 cells expressing IZUMO1 (Inoue et al., 2013; Chalbi et al., 2014; Ohto et al., 2016) or JUNO (Bianchi et al., 2014; Chalbi et al., 2014; Kato et al., 2016) attach to the ZP-free eggs or sperm, respectively, but never fused with them. To better understand the functions of IZUMO1 and JUNO, the plasma membrane fusion needs to be directly investigated using sperm and eggs. However, the ZP-intact eggs are not ideal for studying gamete fusion *in vitro*. With the presence of the ZP, sperm penetration and fusion timing is not controllable. In addition, the cortical reaction is triggered after incorporating the initial spermatozoon with the oolemma, thereby preventing additional spermatozoa from penetrating the ZP and approaching the oolemma. Therefore, the ZP should be removed mechanically by a piezo-driven micromanipulator or chemically by collagenase or acidic Tyrode before the fusion analysis (Yamagata et al., 2002; Inoue et al., 2005; Yamatoya et al., 2011; Nozawa et al., 2018). Nevertheless, the acrosomal-intact sperm, which cannot pass through the ZP or fuse with the oolemma, non-specifically bind to the egg membrane in a fusion-incompetent way in mice and hamsters (Phillips and Yanagimachi, 1982; Calarco, 1991; Yanagimachi, 1994).

Like mice and hamsters, rats have been used as a popular rodent model for spermatogenesis and reproductive toxicology (Russell et al., 1993; OECD, 2016). However, they have not been used intensively in the fertilization field due to a delay in developing a highly efficient and convenient *in vitro* fertilization system (Honda et al., 2019). Distinct to the previous findings using mice or hamsters, we discovered that the acrosome-intact sperm in rats are less likely to adhere to the oolemma than the acrosome-reacted sperm. Such uniqueness in the rats enables us to explicitly quantify the fusion-competent binding between the acrosome-reacted sperm and oolemma. In a previous study, the acrosome-intact human sperm adhered to the mouse egg plasma membrane but not to the human egg surface (Liu and Baker, 1994). Thus, it is tempting to speculate that the fusion-incompetent sperm adhesion in mice and hamsters might be attributed to the molecular architecture of the egg plasma membrane.

In combination with a transgene encoding Acrosin-GFP, the sperm acrosome status can be readily examined by the presence or absence of the green fluorescence signals (Nakanishi et al., 1999; Hasuwa et al., 2010). In 2014, Bianchi et al. reported the adhesion rate of the acrosome-reacted *Acr-*GFP spermatozoa to the JUNO-deficient egg membrane is significantly reduced compared to that of the wild-type group. Here, using a similar approach, we discovered that the IZUMO1-deficient mouse spermatozoa hardly adhere to the egg membrane after the acrosome reaction. However, a small population of the acrosome-reacted *Izumo1* KO mouse spermatozoa can still adhere to the oolemma, which might be attributed to the existence of other sperm-oolemma adhesion machinery on top of the IZUMO1-JUNO interaction in mice.

Interestingly, a more recent study indicates that SPACA6 is absent in the *Izumo1* KO mouse spermatozoa (Inoue et al., 2021). Therefore, it remains possible that the complete loss of the oolemma binding ability in the *Izumo1* KO rat spermatozoa might be attributed to not only the loss of the IZUMO1-JUNO interaction but also the collaterally disrupted interactions between SPACA6 or other sperm surface proteins with their receptors on the egg surface. Therefore, to further investigate the complex functions of those newly identified fusion-related molecules, the fusion-competent adhesion between the Juno KO eggs and the *Fimp*, *Sof1*, *Spaca6*, or *Tmem95* KO spermatozoa that retain IZUMO1 should also be analyzed. It should be noted that, while we are preparing this manuscript, *Dcst1/2* KO spermatozoa were also added to the list (Inoue et al., 2021; Noda et al., 2021).

In conclusion, our data suggest that IZUMO1 is critical for the sperm-egg adhesion preceding the plasma membrane fusion in mice and rats. Rats have been demonstrated as a suitable model for the study of mammalian fertilization because the acrosome-intact spermatozoa are less likely to adhere to the ZP-free egg surface non-specifically. Furthermore, the CRISPR/Cas9 system simplifies genome editing in rats, thereby making the genetically modified rats more accessible for researchers. Collectively, this study highlights that the rat model could be intensively employed for efficient screening of molecules involved in the sperm-egg adhesion and fusion by either genetic approaches or inhibition analyses using recombinant proteins or antibodies.

## Data Availability

The original contributions presented in the study are included in the article/Supplementary Material, further inquiries can be directed to the corresponding author.
