# Supplementary material for: Sperm IZUMO1 Is Required for Binding Preceding Fusion With Oolemma in Mice and Rats
Source: Front Cell Dev Biol. 2022 Jan 12;9:810118. doi: 10.3389/fcell.2021.810118 (PMC8790511; doi:10.3389/fcell.2021.810118)
Supplement: Supplementary file 1 [file DataSheet1.PDF]

Supplementary Material

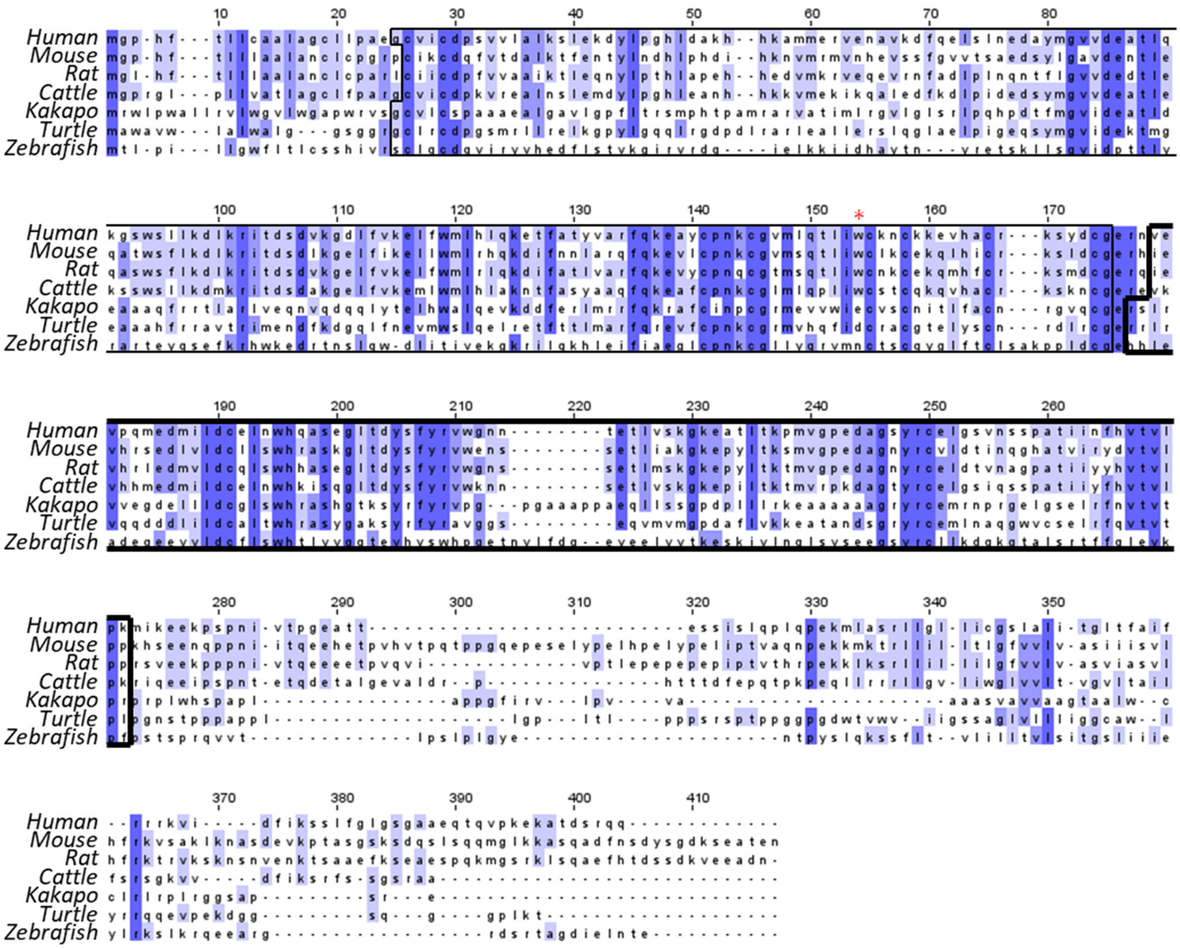

Supplementary Figure 1 | Sequence alignment of IZUMO1 proteins in various organisms.

Sequence similarity of IZUMO1 in *Homo sapiens* (Human), *Mus musculus* (Mouse), *Rattus norvegicus* (Rat), *Bos taurus* (Cattle), *Strigops habroptila* (Kakapo), *Terrapene carolina triunguis* (Turtle), and *Danio rerio* (Zebrafish). Dark blue indicates a sequence match in all the species. Light blue indicates a match among at least three species. Thin and bold black boxes indicate the IZUMO domain and the Ig-like domain, respectively. W148 of human IZUMO1 (red asterisk) is important for interaction with JUNO, and this amino acid residue is conserved in mice, rats, and cows.

A

pX330 (ng/µl)	injected	transferred	born	mutant	*rIzumo1*
5	50	23	2	0	
10	84	40	4	1	*-8/-29* (male)
20	81	47	7	1	mosaic (female)

B *rIzumo1*

	sgRNA / PAM
wt	GGTGGCTGCAATAAAGACTTTGGAGCAGAATTACCTGCCTACCCACCTGG
em1 (-7)	GGTGGCTGCAATAAAGA-------GCAGAATTACCTGCCTACCCACCTGG
em2 (-8)	GGTGGCTGCAAT--------TGGAGCAGAATTACCTGCCTACCCACCTGG
em3 (-29)	GGTGGCTGC-----------------------------CTACCCACCTGG

C **D**

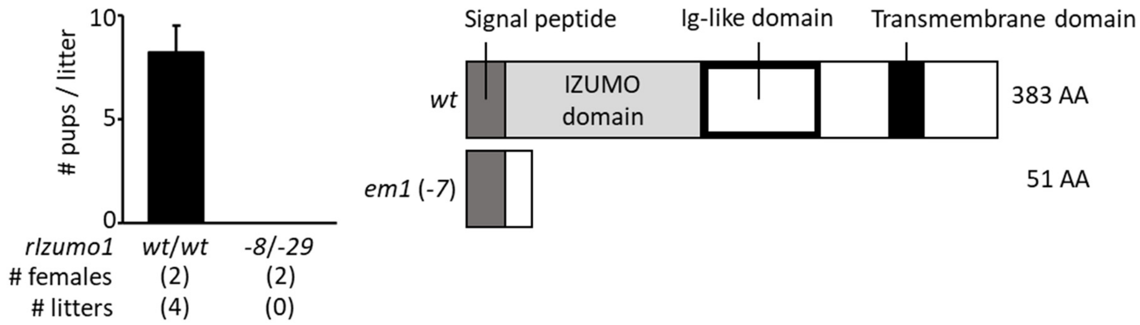

Supplementary Figure 2 | Efficiency of the CRISPR/Cas9-mediated genome editing in rat *Izumo1*.

(A) Efficiency of generating *Izumo1*-deficient rats by injecting different concentrations of the sgRNA/Cas9-encoding plasmid. We injected the sgRNA/CAS9 expressing plasmid (pX330) at 5 ng/µl, 10 ng/µl, or 20 ng/µl into the pronuclei of total 215 zygotes. We obtained a male and female mutant rat from the zygotes injected with pX330 at concentrations of 10 ng/µl and 20 ng/µl, respectively. The *Izumo1* alleles of a mutant founder male (*-8/-29*) are shown in (B). **(B)** The *Izumo1* alleles in the founder animals. The *em1* allele is derived from the mutant founder female. The *em2* and *em3* alleles are derived from the mutant founder male. **(C)** Fertility test of the founder *Izumo1*$^{-8/-29}$ male. **(D)** The expected IZUMO1 protein structures before and after the -7 mutation.

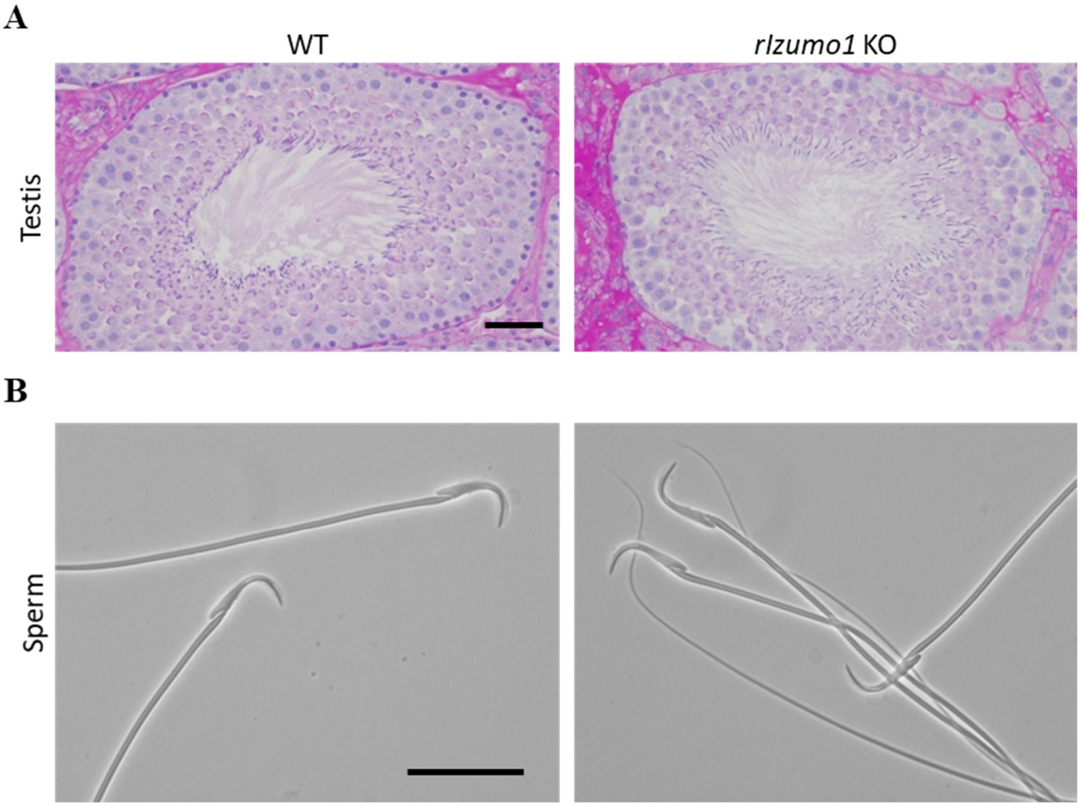

Supplementary Figure 3 | Spermatogenesis in *Izumo1* disrupted rats.

(A) Histology of the adult rat testis. The testis sections were stained with Hematoxylin and periodic acid-Schiff reagents. Scale bar = 50 μm. **(B)** Morphology of the rat sperm. Scale bar = 20 μm.

\

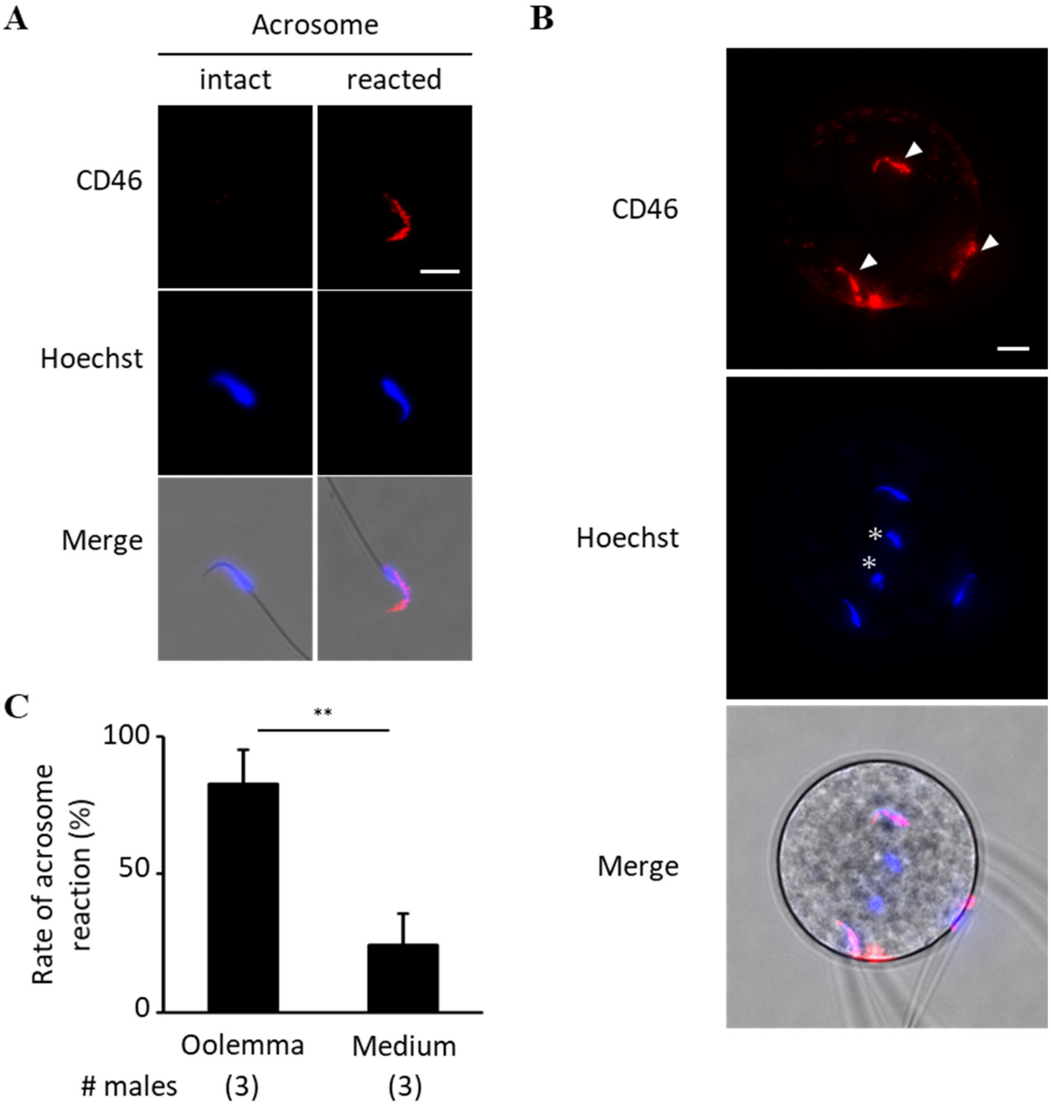

Supplementary Figure 4 | Detection of the sperm acrosome reaction and the binding between the acrosome-reacted sperm and the ZP-free eggs in rats.

(A) Assessment of the spontaneous acrosome reaction. The spontaneous acrosome reaction was assessed using an anti-CD46 antibody. Only the acrosome-reacted sperm were stained. Scale bar = 10 µm. **(B)** The acrosome status of the sperm adhered to the ZP-free rat eggs was examined using the CD46 antibody. The arrowheads indicate acrosome reacted spermatozoa. The asterisks indicate egg chromosomes. Scale bar = 10 µm. **(C)** The acrosome reaction rate of the sperm adhered to the oolemma compared to that of the sperm in the medium. $^{**}p < 0.01$.

Supplementary Movie 1 | *Izumo1* Het rat sperm adhere to the oolemma after 5 hours of insemination

(.mp4)

Supplementary Movie 2 | *Izumo1* KO rat sperm fail to adhere to the oolemma after 5 hours of insemination

(.mp4)
